# Colorectal cancer molecular subtypes and prognostic models based on NET-related genes and oxidative stress-associated signaling

**DOI:** 10.1097/MD.0000000000050211

**Published:** 2026-08-14

**Authors:** Yi Wei, WeiJian Chu, ChunHui Rao, Yan Yang

**Affiliations:** a Department of Proctology, Hangzhou TCM Hospital Affiliated to Zhejiang Chinese Medical University, Hangzhou, Zhejiang, China; b Department of Otolaryngology, Banshan Community Health Service Center, Gongshu District, Hangzhou, Zhejiang, China.

**Keywords:** colorectal cancer, molecular subtypes, neutrophil extracellular traps, oxidative stress, prognostic risk model

## Abstract

Colorectal cancer (CRC) exhibits marked molecular and microenvironmental heterogeneity, and the TNM staging system alone does not fully explain differences in prognosis or treatment response. Neutrophil extracellular traps (NETs) and oxidative stress participate in inflammatory remodeling, immune regulation, and tumor progression in CRC. However, transcriptomic studies integrating NET-related programs with oxidative stress-associated signaling for molecular stratification and prognostic modeling remain limited. Transcriptome and clinical data from TCGA-COADREAD (training set, 449 tumor and 39 normal samples) and GSE39582 (validation set, 566 tumor and 19 non-tumor samples) were analyzed. Candidate NET-related and oxidative stress-associated genes were collected from GeneCards, Gene Ontology, and published NETosis signatures, and were further restricted by differential expression analysis (|log_2_FC| > 0.585, adjusted *P* < .05) and univariate Cox analysis (*P* < .05). Consensus clustering, survival analysis, immune infiltration analysis, mutation analysis, and oncoPredict-based drug sensitivity modeling were performed. A multigene prognostic model was constructed using LASSO-Cox regression and evaluated in both cohorts. Two molecular subtypes were identified. C1 showed a better prognosis, higher tumor mutational burden, higher immune checkpoint and immunogenicity scores, and an immune-active phenotype. C2 showed poorer survival, enrichment of NET formation and inflammatory pathways, lower relative neutrophil infiltration by CIBERSORT, and predicted sensitivity to JAK, Wnt/β-catenin, proteasome, and cell-cycle checkpoint inhibitors. The prognostic model separated high- and low-risk patients in both the training and validation cohorts, with 1-, 3-, and 5-year area under the curves of 0.698, 0.711, and 0.645 in TCGA-COADREAD and 0.542, 0.578, and 0.569 in GSE39582. This study proposes NET-related CRC molecular subtypes with oxidative stress-associated biological context and develops a transcriptomic risk model with external validation. The findings suggest subtype-specific immune and therapeutic features, while prospective clinical and functional validation is required before clinical translation.

## 1. Introduction

Colorectal cancer (CRC) is one of the most common malignant tumors worldwide, with persistently high incidence and mortality rates.^[1]^ It is predicted that the number of new CRC cases globally will more than double by 2035.^[2,3]^ CRC is highly heterogeneous at the genomic, transcriptomic, and microenvironmental levels, and this heterogeneity contributes to variable clinical outcomes and treatment sensitivity.^[4,5]^ Despite advances in surgery, chemotherapy, targeted therapy, and immunotherapy, a substantial proportion of patients still experience recurrence or progression, underscoring the need for biologically informative stratification strategies.^[6,7]^

The TNM staging system remains the main basis for prognosis assessment and treatment decision-making in CRC.^[8-10]^ However, patients with the same anatomical stage may have markedly different survival outcomes and therapeutic responses.^[11,12]^ This limitation reflects the fact that tumor behavior is shaped not only by local invasion and metastasis but also by tumor-intrinsic molecular programs, immune infiltration, stromal remodeling, and inflammatory signaling within the tumor microenvironment (TME).^[13,14]^ Therefore, molecular biomarkers that complement established clinicopathological variables may improve patient risk stratification and provide a basis for individualized treatment.

Transcriptome-based molecular classification has become an important approach for characterizing CRC heterogeneity.^[15,16]^ The consensus molecular subtype (CMS) system classifies CRC into 4 major subtypes with distinct immune, epithelial, metabolic, and mesenchymal features.^[6]^ The CMS framework is well established and is not replaced by the present study. Instead, our work focuses on a specific biological axis, namely neutrophil extracellular trap (NET)-related inflammation and oxidative stress-associated signaling, to explore whether this axis can further define clinically meaningful differences within CRC and provide a mechanistic layer that complements CMS classification.

Among TME components, neutrophils and their derived NETs have become a focus of CRC research.^[17-21]^ NETs consist of decondensed chromatin decorated with granular proteins such as neutrophil elastase and myeloperoxidase, and are released during NETosis in response to inflammatory, microbial, or tumor-derived stimuli.^[22,23]^ In cancer, NETs may promote tumor cell adhesion, invasion, immune escape, and metastasis.^[17,24]^ NETosis is closely connected with reactive oxygen species, because nicotinamide adenine dinucleotide phosphate oxidase-dependent reactive oxygen species production can initiate downstream histone modification, chromatin decondensation, and extracellular chromatin release.^[25,26]^ Thus, NET-related genes and oxidative stress-associated genes may jointly reflect inflammatory and redox states in CRC.

Recent studies have shown that immune receptor signaling, cytokine-driven inflammatory responses,^[27]^ infection-associated immune remodeling,^[28]^ and protein conformational regulation are closely related to immune activation and tissue inflammatory status.^[29-31]^ These findings indicate that tumor progression is influenced not only by intrinsic genetic alterations, but also by broader immune and stress-related biological processes. In CRC, such processes may interact with neutrophil activation, oxidative stress, and microenvironmental remodeling, providing a rationale for investigating NET- and oxidative stress-associated transcriptional features in molecular subtyping and prognostic assessment.

Although NETs and oxidative stress have each been implicated in CRC progression, integrated transcriptomic analysis of these processes remains limited. We therefore identified NET-related and oxidative stress-associated prognostic genes, defined CRC molecular subtypes, and characterized their survival, immune infiltration, mutational, and drug-response features. We further constructed a LASSO-Cox risk model to evaluate whether the selected genes could provide additional prognostic information. This study evaluates NET/oxidative stress-related transcriptional features as a complementary layer for CRC stratification and prognostic modeling.

## 2. Materials and methods

### 2.1. Data acquisition

In this study, we downloaded the transcriptome RNA sequencing data (a total of 514 samples) of the CRC project (TCGA-COADREAD) and the corresponding clinical follow-up information from https://xenabrowser.net/datapages/?dataset=TCGA-COAD.star_counts.tsv&host=https%3A%2F%2Fgdc.xenahubs.net&removeHub=https%3A%2F%2Fxena.treehouse.gi.ucsc.edu%3A443. We excluded samples with missing survival time, survival status, or key clinical information, and ultimately obtained 449 tumor samples and 39 normal samples as the training set for this study. Meanwhile, we downloaded the GSE39582 dataset from the GEO (Gene Expression Omnibus) database (https://www.ncbi.nlm.nih.gov/geo/) as an external validation cohort (566 CRC tumor tissue samples and 19 non-tumor tissue samples).

The gene sets related to NETs and oxidative stress were obtained from GeneCards (https://www.genecards.org/), Gene Ontology (https://geneontology.org), and published NETosis-related literature. GeneCards-derived genes were used as a broad discovery background. To reduce noise from this broad candidate pool, genes were required to pass downstream transcriptomic and prognostic filters before entering subtype construction. The NET-related pool included 2569 GeneCards genes and 69 genes reported by Qi et al, yielding 2601 nonredundant NET-related candidates. Oxidative stress-associated genes were collected from GeneCards and Gene Ontology, yielding 564 candidates.

### 2.2. Determination of the core prognostic gene set

First, in the TCGA-COADREAD cohort, the R package survival was used to perform univariate Cox regression analysis to screen genes associated with overall survival (OS; *P* < .05). DESeq2 was then used to compare tumor and adjacent normal samples, with |log_2_(fold change)| > 0.585 and adjusted *P* < .05 as the differential expression threshold. The intersection of prognostic genes, differentially expressed genes (DEGs), and the NET/oxidative stress candidate pool was defined as the core prognostic gene set. This strategy required both statistical association and biological annotation, thereby avoiding reliance on database membership alone.

### 2.3. Construction and characterization of CRC molecular subtypes

We used the R package ConsensusClusterPlus to perform consensus clustering analysis on the expression matrix of the core gene set in the TCGA tumor cohort. The optimal cluster number was determined by evaluating the cumulative distribution function (CDF), Delta Area plot, and the clarity of the consensus matrix. Survival analysis was conducted with Kaplan–Meier curves, and OS differences were compared using the log-rank test. Gene set enrichment analysis (GSEA) and gene set variation analysis (GSVA) were used to evaluate pathway differences between subtypes. CIBERSORT was used to estimate the relative abundance of 22 immune cell types, and immune checkpoint gene expression and immunogenicity score (IPS) were compared between subtypes. Somatic mutation data were analyzed using maftools to calculate tumor mutational burden (TMB) and generate mutation spectra. Drug sensitivity was estimated using oncoPredict, which builds ridge regression models from cancer cell line expression and drug-response datasets and then imputes drug response in tumor transcriptomes.^[32]^ Predicted half maximal inhibitory concentration (IC50) values were compared between subtypes; lower predicted IC50 was interpreted as higher modeled sensitivity.

### 2.4. Construction of prognostic risk model

Based on the core gene set, a multigene prognostic risk model was constructed. Univariate Cox regression first screened OS-associated genes, and LASSO-Cox regression with 10-fold cross-validation was then used to reduce overfitting and determine the optimal lambda. The final model retained 8 genes (PTH1R, FOXC1, CALB1, H2BC21, CDKN2A, CPT1B, DYNC1I1, and SLC4A4). Each patient’s risk score was calculated as the sum of the standardized expression level of each retained gene multiplied by its Cox regression coefficient.

### 2.5. Evaluation and validation of the risk model

In the TCGA training set, patients were divided into high- and low-risk groups according to the median risk score. Kaplan–Meier survival curves, risk distribution plots, and time-dependent receiver operating characteristic curves were used to evaluate model performance. Multivariate Cox analysis was used to assess whether the risk score retained prognostic value after adjustment for available clinical factors, including age, sex, and TNM/pathologic stage. Microsatellite instability (MSI) status was considered when available; however, incomplete annotation across samples limited its use in cross-cohort comparisons.

### 2.6. External validation of the risk model

To verify model transferability, the training set-derived formula was applied to GSE39582 after within-cohort standardization. The training set median risk score was used as the cutoff for risk stratification. Because TCGA-COADREAD is an RNA-seq cohort whereas GSE39582 is a microarray cohort, the 2 datasets were not merged for joint modeling.

### 2.7. Statistical analysis

All data were analyzed and visualized in RStudio. Continuous variables between 2 groups were compared using the Wilcoxon rank-sum test, and categorical variables were compared using the chi-square test or Fisher exact test when appropriate. Survival analysis used Kaplan–Meier curves and log-rank tests. Cox proportional hazards models were used for univariate and multivariate survival analyses, and hazard ratios with 95% confidence intervals were reported where applicable. *P* < .05 was considered statistically significant.

## 3. Results

**Figure 1.**
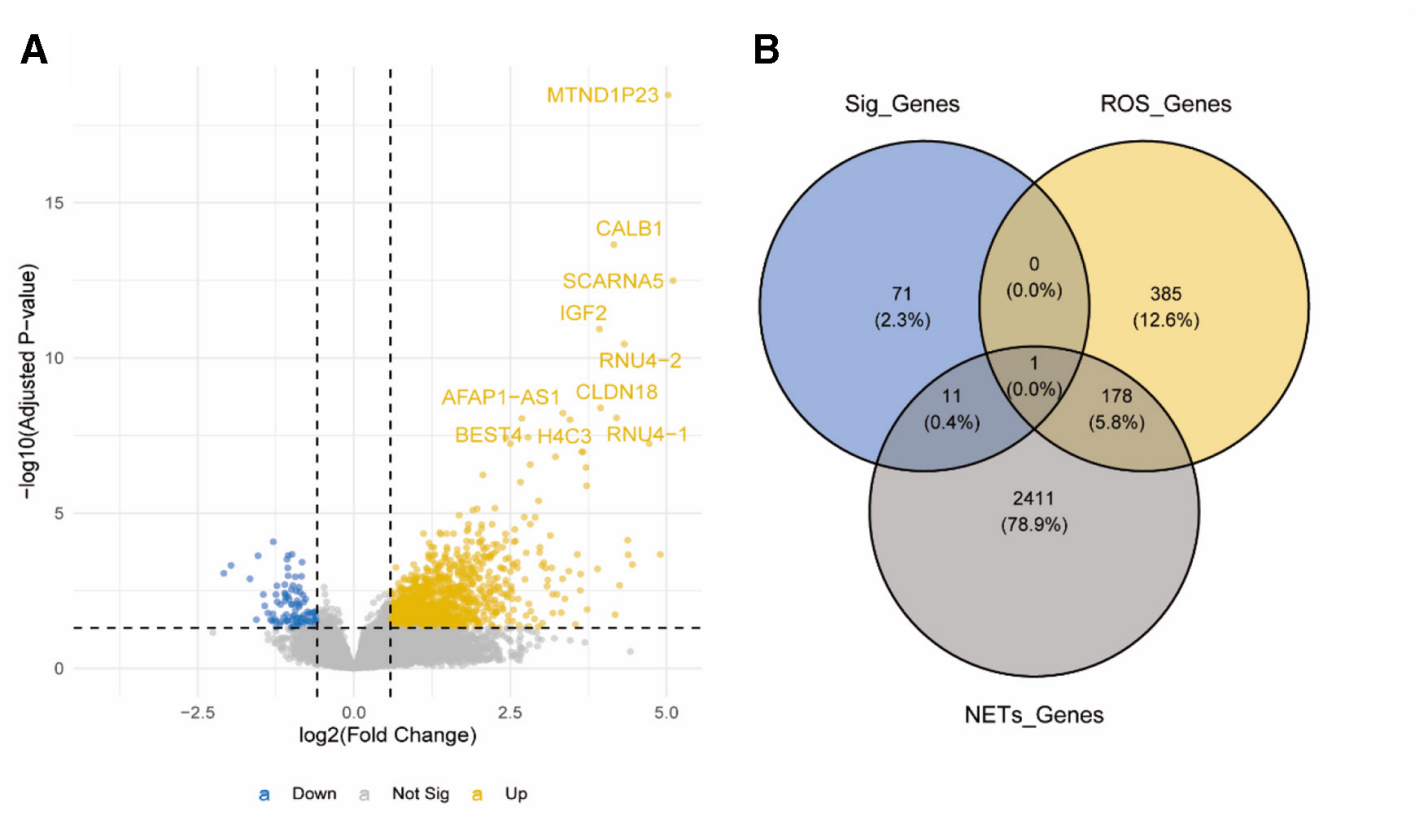
Core prognostic gene screening strategy. (A) DEGs between tumor and normal samples: yellow/blue dots = significantly up/downregulated genes in tumors; gray dots = nonsignificant genes. (B) Venn diagram of core prognostic genes: intersections of NETs-related, oxidative stress-related, and univariate Cox prognostic genes (numbers = gene count and percentage of total). DEGs = differentially expressed genes, NET = neutrophil extracellular trap, ROS = reactive oxygen species.

**Figure 2.**
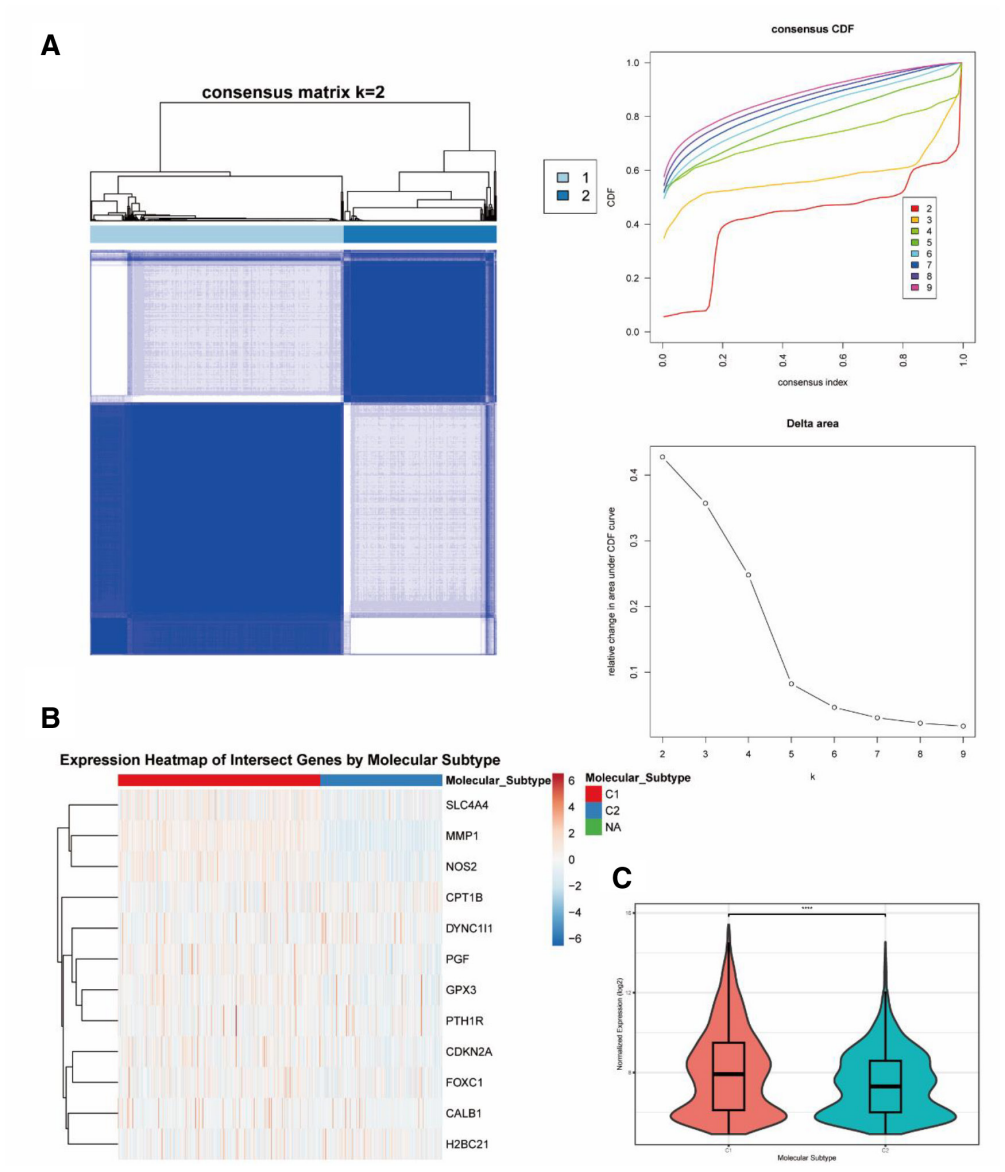
Molecular subtype clustering and characteristic expression based on the core prognostic gene set in colorectal cancer. (A) Consensus clustering analysis determined the optimal clustering type (*k* = 2). (B) The clustering heatmap shows the core genes between the C1 and C2 molecular subtypes. (C) The violin plot shows the overall expression level differences of the core genes between the 2 subtypes, where represents *P*-value < .001. CDF = cumulative distribution function.

To screen core DEGs related to prognosis, we first performed differential expression analysis between CRC tumor and normal tissue samples in the TCGA training set (Fig. 1A). We then performed univariate Cox regression analysis on genes associated with NETs and oxidative stress to identify genes significantly related to OS. Intersecting the DEG set, prognostic gene set, and NET/oxidative stress candidate pool yielded 12 core prognostic feature genes: SLC4A4, MMP1, NOS2, CPT1B, DYNC1I1, PGF, GPX3, PTH1R, CDKN2A, FOXC1, CALB1, and H2BC21 (Figs. 1B and 2B). Eleven genes were annotated as NET-related, and 1 gene overlapped both NET-related and oxidative stress-associated sets. The signature was therefore dominated by NET-related genes, with oxidative stress represented by 1 overlapping gene.

Biologically, these genes were considered relevant to subtype construction because they cover multiple processes through which NETs and oxidative stress may influence CRC. MMP1 and H2BC21 are linked to matrix degradation and chromatin-related events, NOS2 and GPX3 represent redox and antioxidant regulation, PGF contributes to angiogenesis and inflammatory cell recruitment, CDKN2A reflects stress-induced cell-cycle arrest, FOXC1 and PTH1R have been associated with invasive and stromal signaling programs, and SLC4A4, CPT1B, DYNC1I1, and CALB1 reflect epithelial transport, metabolic adaptation, intracellular trafficking, and calcium signaling. Thus, the 12 genes were used not as canonical NETosis markers alone but as a prognostic transcriptomic signature related to NET-associated inflammation and oxidative stress biology.

To explore prognostic molecular subtypes, unsupervised consensus clustering was performed using the expression matrix of the 12 core genes. The cumulative distribution function curve, Delta Area plot, and consensus matrix supported *k* = 2 as the most stable and interpretable clustering scheme, classifying patients into C1 and C2 subtypes (Fig. 2A). The *k* = 2 solution captured a focused NET/oxidative stress-related axis within CRC. Heatmap and violin plot analyses showed significant differences in the overall expression pattern of the core genes between subtypes (*P* < .001), including SLC4A4, MMP1, NOS2, PTH1R, FOXC1, and CALB1 (Fig. 2B, C).

**Figure 3.**
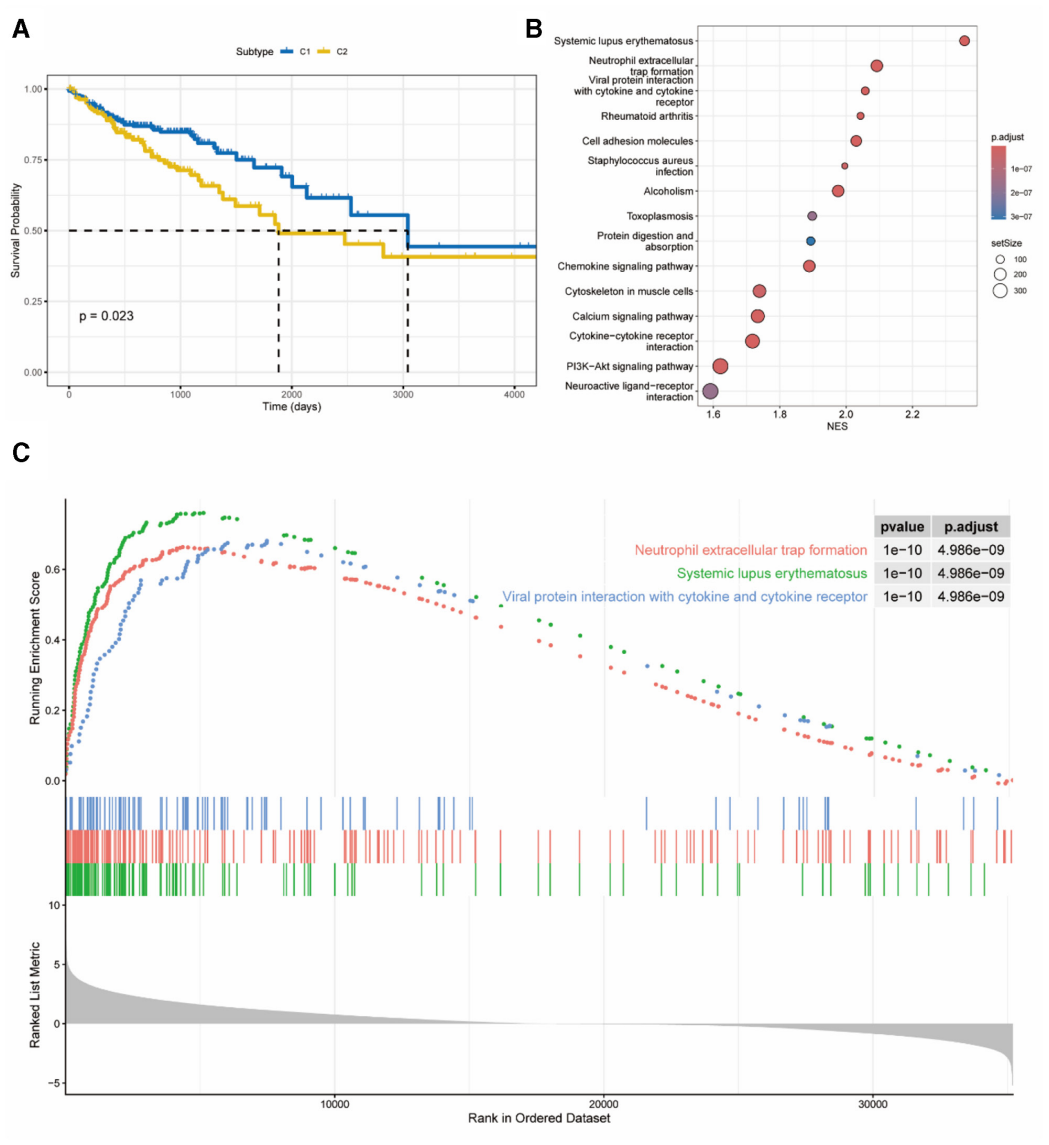
Clinical prognosis and functional enrichment of 2 CRC molecular subtypes C1 and C2. (A) Kaplan–Meier survival curves for CRC subtypes classified by core signature genes; dashed lines indicate the median survival of each group, and log‐rank statistics were used to evaluate survival discrepancies between C1 and C2 subtypes. (B) Bubble chart showing significantly enriched pathways uniquely activated in C2 subtype via GSEA. X‐axis: NES; Y‐axis: annotated pathway names. Bubble size stands for the quantity of enriched genes in each pathway, and color depth corresponds to FDR‐adjusted *P* value to reflect statistical significance. (C) GSEA enrichment diagrams of the top 3 prominent pathways of C2. Vertical barcodes exhibit the distribution of pathway genes in the ranked gene list, and the dot curve illustrates the cumulative enrichment score change across the gene ranking sequence. CRC = colorectal cancer, GSEA = gene set enrichment analysis, NES = normalized enrichment score.

Kaplan–Meier survival analysis showed a statistically significant but moderate OS difference between the C1 and C2 subtypes (*P* = .023). C1 had a better prognosis, whereas C2 showed shorter survival (Fig. 3A). The NET/oxidative stress-related gene set therefore separated a C2 subgroup with poorer survival.

GSEA of the C2 subtype revealed significant activation of inflammation- and immunity-related pathways, including systemic lupus erythematosus, NET formation, and chemokine signaling (Fig. 3B). Enrichment plots (Fig. 3C) confirmed higher pathway-level activity in C2, linking this subtype to inflammatory activation and altered neutrophil functional states.

**Figure 4.**
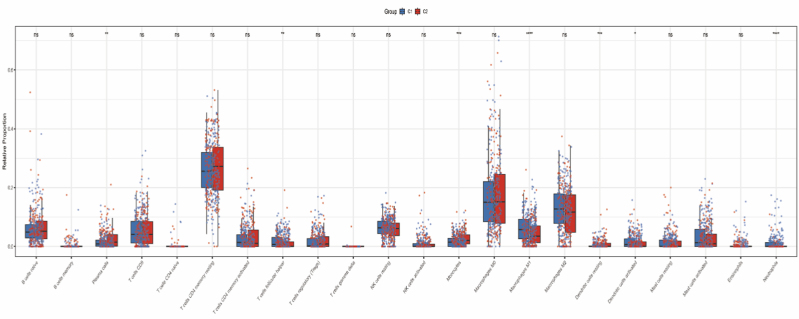
Tumor immune microenvironment infiltration (CIBERSORT). Box plots show relative infiltration of 22 immune cell subtypes in C1/C2. Differential immune microenvironment patterns are revealed by inter-subtype comparisons. Asterisks indicate significance: *P* < .05, *P* < .01, *P* < .001; ns = nonsignificant.

CIBERSORT analysis (Fig. 4) revealed distinct immune cell profiles between C1 and C2. C1 had higher relative fractions of neutrophils (*P* < .001), M1 macrophages (*P* < .001), follicular helper T cells (*P* < .01), and activated dendritic cells, consistent with an immune-active phenotype. C2 showed higher relative fractions of resting dendritic cells, monocytes, and plasma cells (all *P* < .05), suggesting relatively weaker antigen presentation and T-cell activation. CIBERSORT estimates relative cell fractions; absolute neutrophil density was not measured.

**Figure 5.**
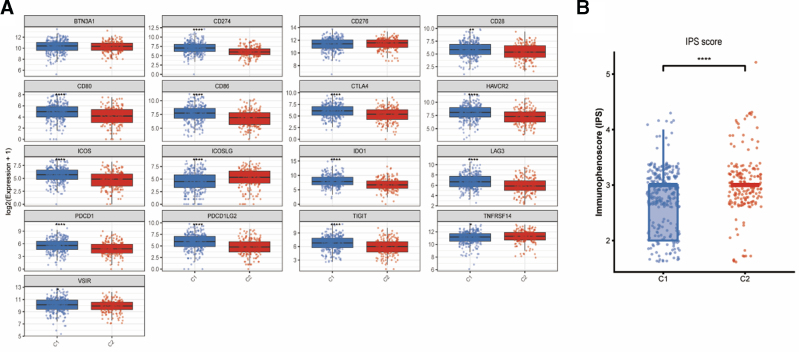
Differences in immune checkpoint expression and immunogenicity score (IPS) between C1 and C2 subtypes. (A) Figure shows the expression level differences of key immune checkpoint genes (ICGs) between C1 and C2 subtypes. (B) Figure shows the comparison of immunogenicity between C1 and C2 subtypes. Here, * represents *P* < .05, ** represents *P* < .01, *** represents *P* < .001, and **** represents *P* < .0001.

To assess immune checkpoint inhibitor (ICI) efficacy differences between subtypes, we compared key immune checkpoint gene (ICG) expression. Results (Fig. 5A) showed significant ICG expression disparities: favorable-prognosis C1 had higher PDCD1, CTLA4, and LAG3 levels than C2, indicating C1 may be in “adaptive immune resistance” with active antitumor immunity.

We further performed IPS (based on MHC/EC/SC/CP) analysis. Results (Fig. 5B) showed C1 had significantly higher IPS than C2 (*P* < .05), suggesting C1 has “hot tumor” characteristics and higher ICI sensitivity, while C2 is a “cold tumor” with potential natural ICI resistance.

**Figure 6.**
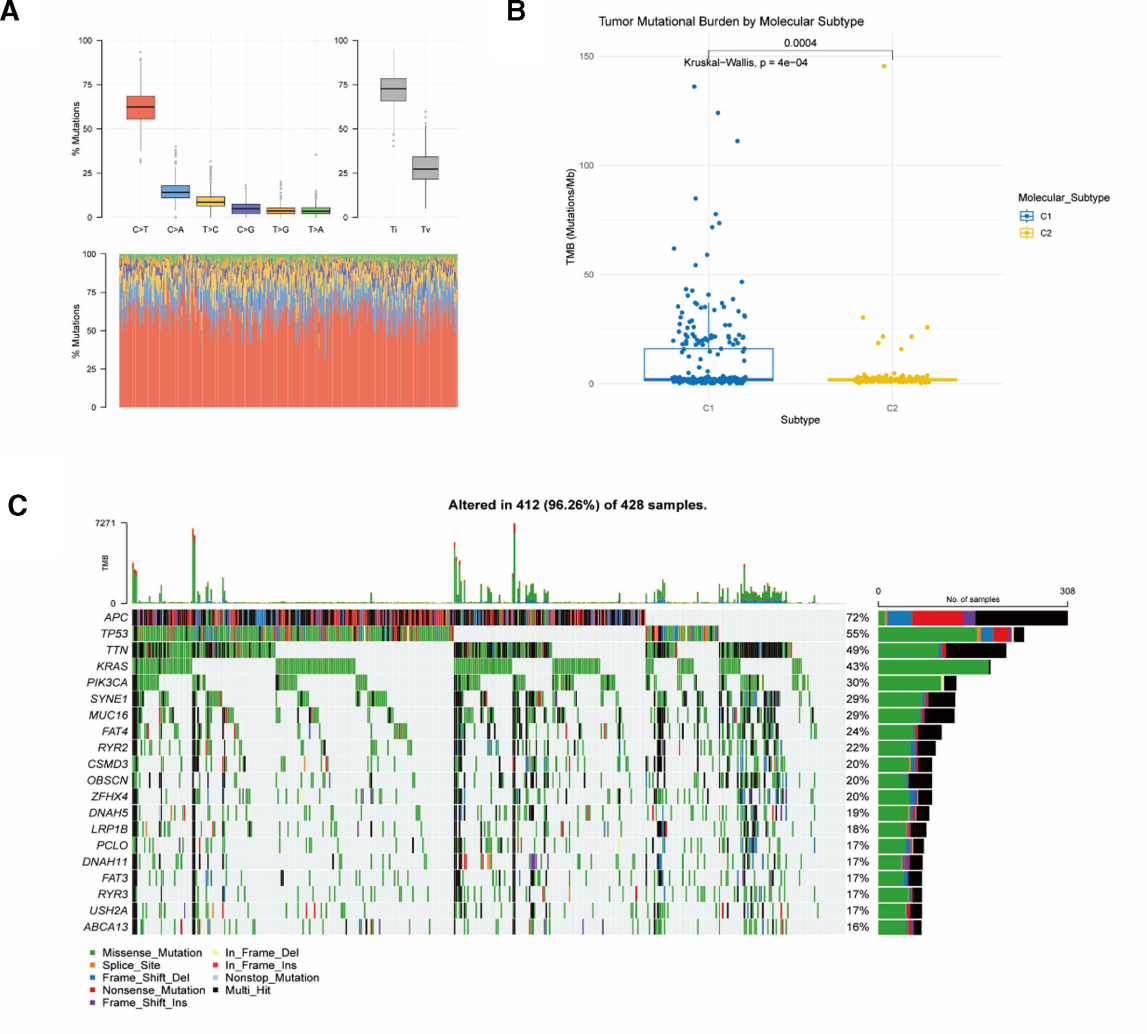
Genomic mutation landscape of subtypes. (A) Tumor mutational signature: top box plot = 6 SNV types and Ti/Tv ratio; bottom stacked bar plot = sample-level mutation type composition. (B) TMB comparison (box plot = mutations per Mb) between C1/C2. (C) Oncoplot: mutation status of top 20 mutated genes (top bar = sample TMB; right bar = gene mutation frequency/type). SNV = single nucleotide variant, TMB = tumor mutational burden.

To explore upstream mechanisms of immunogenicity differences, we analyzed genomic mutations. Most samples had dominant C > T single nucleotide variant substitution and higher Ti than Tv rates (typical CRC feature, linked to aging/mismatch repair deficient; Fig. 6A).

Tumor mutational burden was significantly higher in C1 than in C2 (Fig. 6B), which may contribute to the immune-active phenotype of C1 by increasing neoantigen load. Oncoplot analysis (Fig. 6C) showed that 96.26% of 428 samples had mutations, with APC (72%), TP53 (55%), TTN (49%), KRAS (43%), and PIK3CA (30%) among the most frequently mutated genes, consistent with classic CRC genomic features.

**Figure 7.**
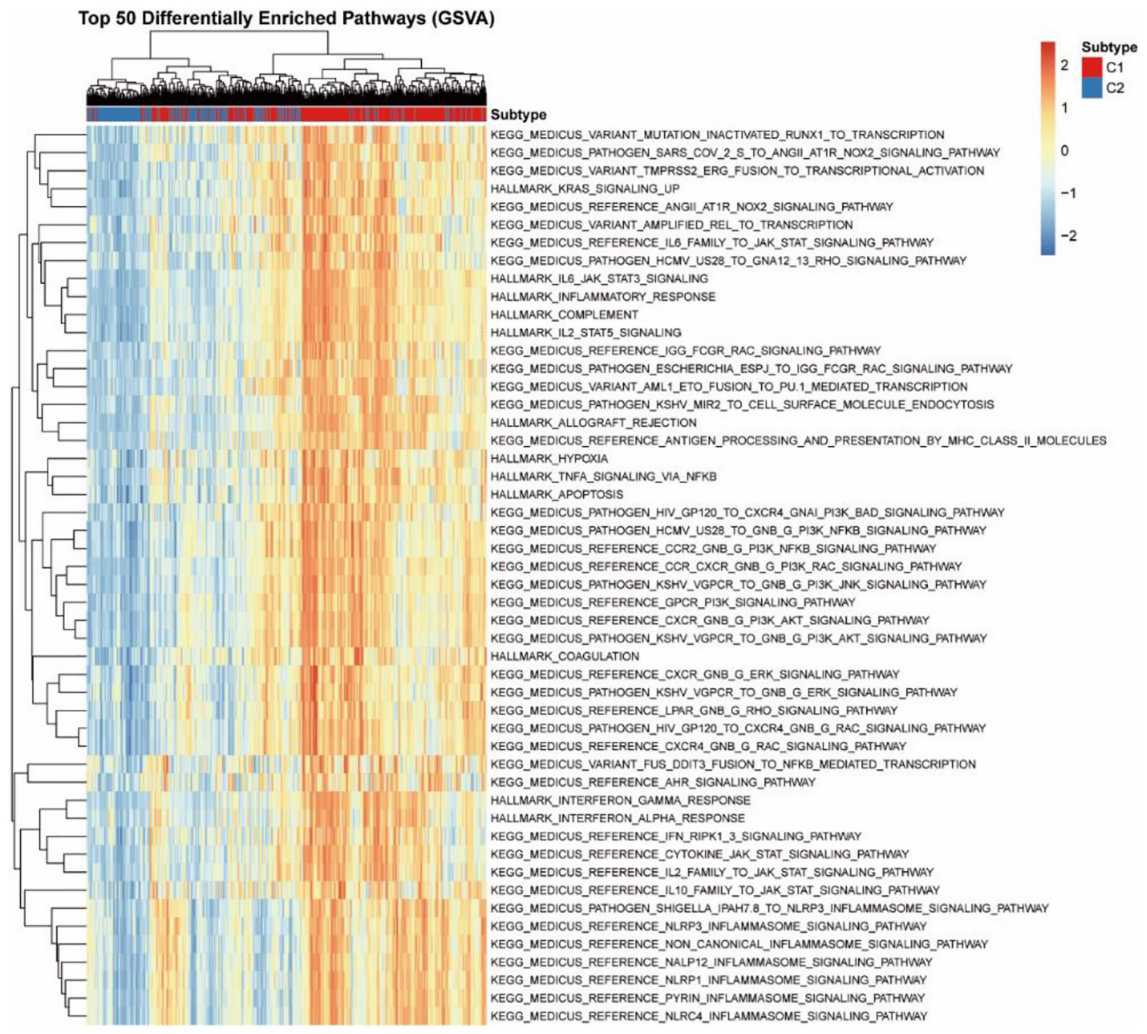
Heatmap of GSVA enrichment scores for biological pathways in colorectal cancer C1 and C2 subtypes. This heatmap exhibits the top 50 pathways with remarkable differential enrichment between the 2 molecular subgroups. Each row represents a single functional pathway collected from Hallmark and KEGG databases, and each column stands for an individual tumor sample grouped by CRC subtype. Cell color intensity mirrors normalized GSVA activity scores: red marks upregulated pathway activation, blue indicates pathway inhibition. Hierarchical clustering of pathways and samples was performed to group samples with consistent functional signatures and identify subtype‐specific biological programs. CRC = colorectal cancer, GSVA = gene set variation analysis.

GSVA analyzed pathway activation differences between C1/C2 subtypes (Fig. 7). Favorable-prognosis C1 showed extensive pathway upregulation: oncogenic signals (e.g., KRAS, IL-6/JAK/STAT3), pro-inflammatory pathways, complement/IL-2/STAT5 signaling, and strong interferon (α/γ) responses. C1 had overall immune enrichment, while C2 was less active – consistent with IPS results.

**Figure 8.**
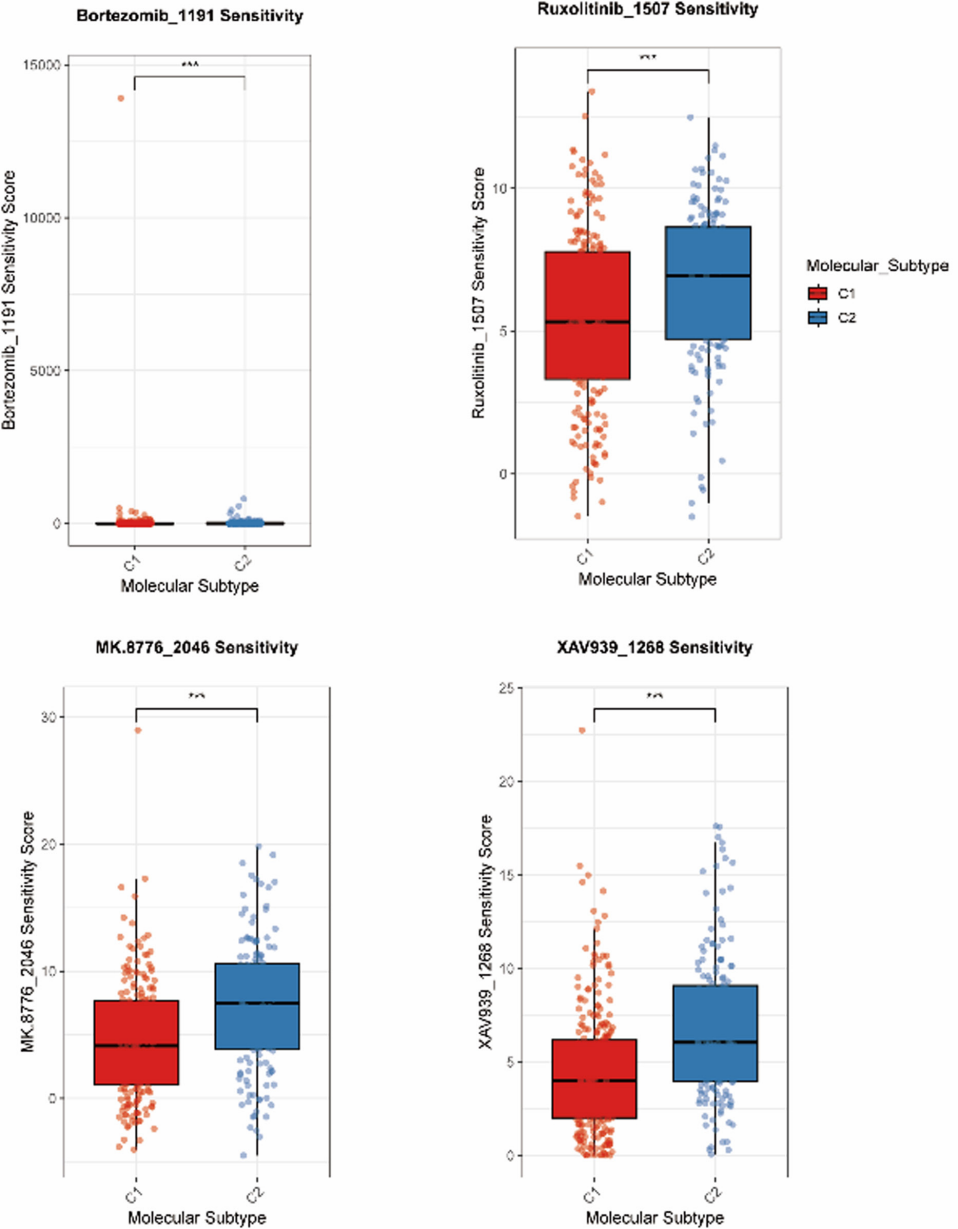
Sensitivity analysis of different subtypes. This box plot compares the predicted sensitivity of C1 and C2 subtypes to a series of targeted drugs and chemotherapy drugs. indicates significant differences between different subtype groups.

Based on GSVA-revealed functional heterogeneity, oncoPredict was used to estimate C1/C2 sensitivity to targeted and chemotherapeutic drugs (Fig. 8). C2 showed lower predicted IC50 values for JAK inhibition (Ruxolitinib_1507), Wnt/β-catenin inhibition (XAV939_1268), proteasome inhibition (Bortezomib_1191), and CHK1/WEE1-related cell-cycle checkpoint inhibition (MK.8776_2046).

**Figure 9.**
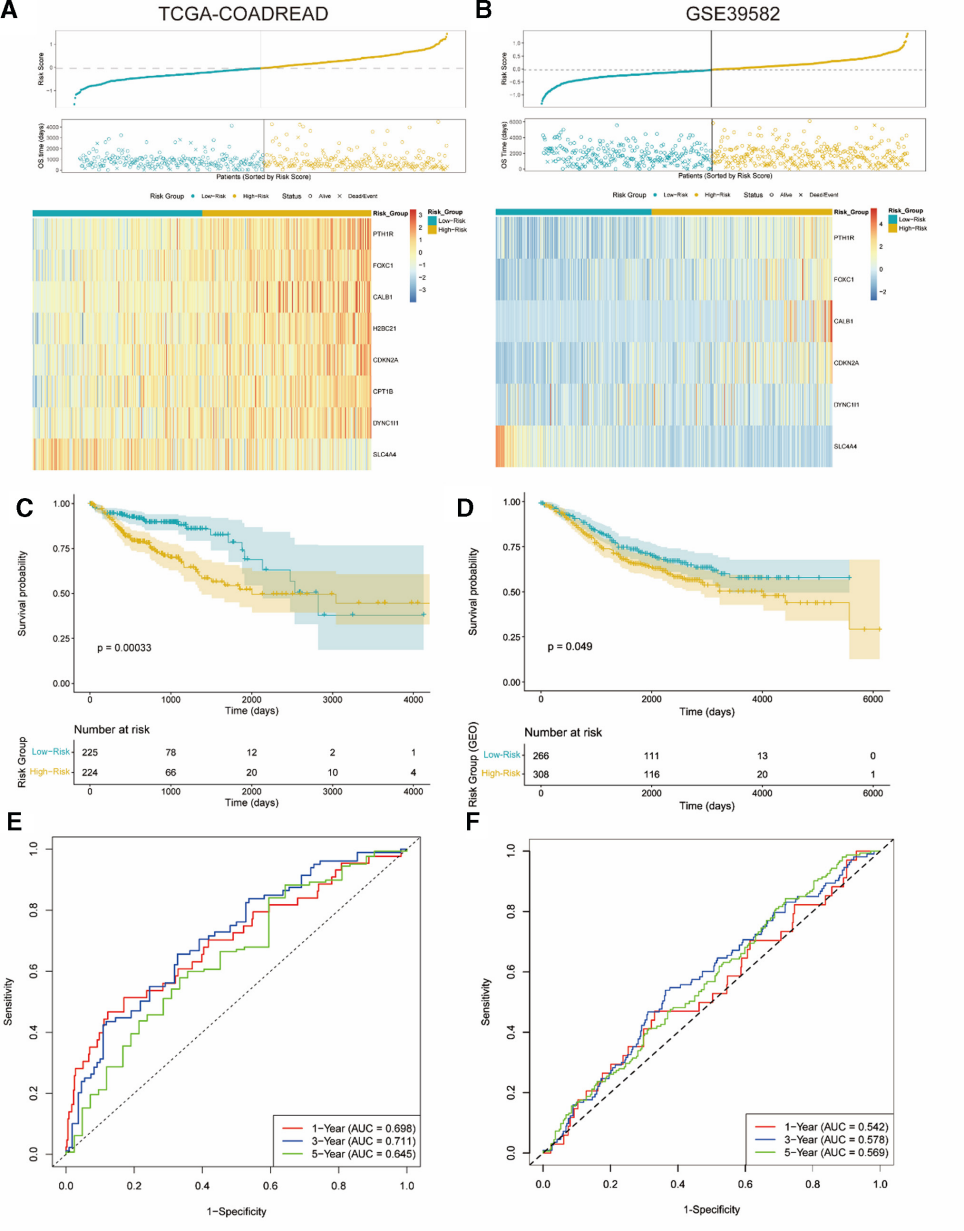
Risk model training and validation. (A, B) Risk factor plots (TCGA/GEO): top = risk score distribution, middle = survival status (dots), bottom = model gene expression heatmap. (C, D) KM survival curves (TCGA/GEO) comparing high/low-risk overall survival. (E, F) Time-dependent ROC curves (TCGA/GEO) for 1-/3-/5-year OS prediction (AUC). AUC = area under the curve, GEO = gene expression omnibus, OS = overall survival, ROC = receiver operating characteristic, TCGA = The Cancer Genome Atlas.

To quantify the prognostic role of the core genes, we constructed a multigene risk model using univariate Cox regression followed by LASSO-Cox regression. In the TCGA training set, patients were stratified into high- and low-risk groups by the median risk score (Fig. 9A). High-risk patients showed higher expression of several risk-associated genes, including PTH1R and FOXC1, and had more death events, whereas SLC4A4 was relatively enriched in the low-risk group.

Kaplan–Meier survival analysis (Fig. 9B) confirmed significantly worse OS in the high-risk group (*P* < .001), supporting the prognostic stratification ability of the model. Time-dependent receiver operating characteristic analysis (Fig. 9C) showed 1-, 3-, and 5-year area under the curves (AUCs) of 0.698, 0.711, and 0.645, respectively.

External validation in GSE39582 (Fig. 9D–F) produced consistent survival separation, with high-risk patients having poorer OS (*P* = .049). The validation AUCs were modest (1-year AUC = 0.542, 3-year AUC = 0.578, 5-year AUC = 0.569), indicating limited cross-platform discriminative performance.

Multivariate Cox analysis further supported the prognostic relevance of the risk score after adjustment for available clinical variables, including age, sex, and TNM/pathologic stage. MSI status was incompletely annotated across the combined analyses and was therefore not used as a uniform adjustment factor in all models.

To clarify the relationship with CMS classification, we compared the biological features of C1/C2 with published CMS characteristics. C1 showed CMS1-like features, including higher immunogenicity and TMB, whereas C2 showed CMS4-like features, including inflammatory pathway enrichment and poor prognosis. Sample-level CMS labels were not uniformly available in the working datasets, so the CMS comparison was based on phenotype-level concordance.

## 4. Discussion

In this study, NET-related and oxidative stress-associated genes were used to identify molecular subtypes and construct a prognostic model. The final 12-gene set was not selected only by statistical association; it also reflects several biological programs relevant to CRC progression, including epithelial ion transport (SLC4A4), extracellular matrix remodeling (MMP1), nitric oxide and inflammatory redox signaling (NOS2), fatty acid oxidation (CPT1B), intracellular transport (DYNC1I1), angiogenic signaling (PGF), antioxidant defense (GPX3), hormone receptor signaling (PTH1R), cell-cycle control (CDKN2A), transcriptional regulation of invasion and epithelial–mesenchymal transition (FOXC1), calcium signaling (CALB1), and chromatin organization (H2BC21). These functions are consistent with the inflammatory, metabolic, and chromatin-related processes that accompany NET formation and oxidative stress in the TME.^[17,22,26,33]^

An important observation was that C2 was enriched in the NET formation pathway but showed lower relative neutrophil infiltration by CIBERSORT. This apparent discrepancy may reflect the difference between neutrophil quantity and neutrophil functional state. NETosis is a terminal or highly activated state in which neutrophils release chromatin and granular proteins; therefore, strong NET-related transcriptional signals may coexist with reduced intact neutrophil fractions in deconvolution analysis. Another explanation is that tumor, stromal, or inflammatory cells may express NET-associated pathway genes, producing a NET-like inflammatory signature without a high abundance of intact neutrophils.

Future experimental validation should therefore examine NETosis-specific markers, including citrullinated histone H3, myeloperoxidase–DNA complexes, and neutrophil elastase, together with neutrophil density markers. Such assays would help determine whether C2 contains fewer neutrophils, more NETotic neutrophils, or a broader NET-like inflammatory transcriptomic program.

C1 displayed higher M1 macrophage and activated dendritic cell fractions, higher TMB, and higher IPS, supporting an immune-active phenotype. Although NETs are often considered pro-tumor factors,^[17,22]^ the biological consequence of NET-related signaling may depend on the accompanying immune and genomic context. In C1, higher neoantigen burden and immune activation may counterbalance oncogenic pathway activity, whereas C2 appears to represent an inflammatory but less effectively immunogenic state. These findings are consistent with the higher immune checkpoint gene expression and IPS observed in C1.

The higher TMB in C1 may increase neoantigen availability and promote antigen presentation, providing a plausible basis for the enrichment of activated dendritic cells and M1 macrophages. In contrast, C2 showed lower TMB and relatively higher resting immune cell fractions, consistent with a less immunogenic or immune-silent phenotype. Nevertheless, MSI status and other clinicopathological variables were not uniformly available across all analyses; therefore, the TMB and immunotherapy-related findings require validation in cohorts with complete MSI annotation and actual immunotherapy outcomes.

The subtype-specific drug sensitivity analysis showed lower predicted IC50 values for ruxolitinib, XAV939, bortezomib, and MK.8776 in C2. The ruxolitinib result is biologically consistent with the role of JAK/STAT signaling in inflammation, cytokine response, immune suppression, and tumor progression.^[34]^

C2 resembles CMS4 in its poor prognosis and inflammatory/stromal features, while C1 partly resembles CMS1 in its immune-active and high-TMB phenotype.^[6,35,36]^ This similarity supports the complementary nature of the NET/oxidative stress-based classification. Rather than proposing a superior alternative to CMS, our results suggest that NET-related inflammatory and redox programs may help explain part of the biological heterogeneity captured by CMS.

This study has several limitations. First, the NET-related candidate gene pool from GeneCards was broad, and although downstream differential expression and survival filters reduced noise, database-derived annotations cannot prove direct functional involvement. Second, TCGA and GSE39582 were generated on different platforms, and external validation was therefore affected by cross-platform batch effects and partial gene loss. Third, immune infiltration was primarily inferred by CIBERSORT, without independent validation by ssGSEA, xCell, or immunohistochemistry. Fourth, IPS, TMB, and drug sensitivity analyses were surrogate or computational predictions and were not validated using actual ICI response data, clinical drug-response data, or in vitro experiments. Finally, the model did not fully integrate MSI status, CMS labels, treatment information, or multi-omics features, which may explain its modest validation AUCs.

## 5. Conclusion

This study defines 2 CRC molecular subtypes based on a NET-dominant gene set with oxidative stress-associated biological context. C1 is characterized by better prognosis, higher TMB, stronger immune activation, and higher predicted immunogenicity, whereas C2 is characterized by poorer prognosis, enrichment of NET formation and inflammatory pathways, lower relative neutrophil infiltration, and predicted sensitivity to JAK/Wnt/proteasome/checkpoint-targeted agents. These subtypes provide a focused complement to existing CRC classifications such as CMS.

A multigene prognostic model was also constructed and externally validated. The model stratified patients into high- and low-risk groups in both TCGA-COADREAD and GSE39582, with modest validation AUCs and platform-related transferability limits. Further integration of clinical variables, MSI status, CMS labels, multi-omics data, independent immune-deconvolution methods, and NETosis markers such as citrullinated histone H3 may improve the clinical utility of these findings.

## Author contributions

**Conceptualization:** ChunHui Rao, Yan Yang.

**Data curation:** Yi Wei, WeiJian Chu.

**Formal analysis:** Yi Wei, WeiJian Chu.

**Investigation:** Yi Wei.

**Methodology:** ChunHui Rao, Yan Yang.

**Validation:** Yi Wei.

**Writing – original draft:** Yi Wei.

**Writing – review & editing:** Yi Wei.
